# WHICH ASPECTS OF HEALTH PREDICT LATE-LIFE SOCIAL INTEGRATION OVER TIME?

**DOI:** 10.1093/geroni/igz038.2305

**Published:** 2019-11-08

**Authors:** Masahiro Toyama, Masahiro Toyama, Heather R Fuller

**Affiliations:** North Dakota State University, Fargo, North Dakota, United States

## Abstract

Associations between late-life social integration and health have been found to be reciprocal. The present study focuses on the direction of health predicting social integration as it is not yet fully understood how different aspects of health may affect social integration. Using two-wave data from a community-based sample (N = 413, mean age 80 at baseline), the present study investigates whether depressive symptoms, chronic health conditions, functional limitations, and self-rated health independently predicted multiple dimensions of social integration over two years. The results of multiple regression and path analyses indicated that self-rated health was the most consistent predictor for social integration over time as the other health measures predicted no or fewer dimensions of social integration. Subjective perception of health appeared to have greater implications for social integration over time than more objective health symptoms/conditions. These findings highlight the important role of subjective health for maintaining late-life social integration.

